# Some Causes of Failures in Operative Dentistry

**Published:** 1898-04

**Authors:** L. F. Frink

**Affiliations:** Lake City, Florida


### Some Causes of Failures in Operative Dentistry.

BY DR. L. F. FRINK, LAKE CITY, FLORIDA.

Abstract of paper read before Southern Branch of National Dental Association, St. Augustine, Florida, February, 1898.

Chief among these is *dull instruments*, especially dull burrs, causing greater friction, more pain, and endangering the life of the pulp. Sharp excavators or chisels are essential in paring away thin marginal walls, especially at the cervical wall.

Another cause is found in fillings placed in too close proximity to the pulp.

Another is the wrong selection of material, either from yielding to the whim of the patient, in using the plastics when gold would be better, from a desire to complete the work more promptly, or in using gold, where other material is indicated, from the desire for a larger fee. A frequent cause of failure is found in neglect in removing salivary calculus. Dr. Frink quoted Dr. Truman's oft-repeated injunction to his students—"First, examine the mouth; second, clean the teeth; then you can see more clearly what should be the treatment." He concluded his short paper by saying: Let us impress upon the minds of our patients the grand lesson of cleanliness in the mouth, as well as of the body, then we will have fewer failures in operative dentistry.

DISCUSSION.

In the discussion of this paper, Dr. W. E. Walker emphasized the importance of sharp burrs; he said the rule in his office is if you can *see the edge*, lay it aside. When they thus accumulate in sufficient quantity, send them to be sharpened. It requires a good light, and, in some cases, a jeweler's glass, but the test is a rare one. Dr. L. M. Cowardin tries the blade on the thumb nail. Dr. Walker considers his method much less tedious than Dr. Cowardin's. In the Revelation burr it is the cross blade over the top that dulls first. Dr. T. B. Cook's method is, when it refuses to act, throw it away. In his experience the Revelation burrs cut well for a little while, but when they get dull you can't do anything with them; the cross-cut burrs are more satisfactory to him, doing twice the amount of work. As to the choice of material, the *fee* is a great temptation, but the material chosen should be the one that will do the best service in the case in hand. Dr. Chas. Sill's practice, in case of near exposure of the pulp, is to dip a thin slice of gutta-percha in chloroform to dissolve the surface only and make it adhere, laying that in the cavity as a foundation for the filling. He prefers this method to the use of chloro-percha. Dr. S. W. Foster considers poorly-prepared margins responsible for more failures than any other one cause. The material employed is of minor importance, provided that the margins are properly prepared. To insure good margins, free separation is necessary. Dr. H. H. Johnson advocates Fissure cross-cut burrs. From 1890 until very recently, the best quality was made only in Germany.

Dr. L. M. Cowardin said that in his early practice he was in the habit of leaving a layer of semi-devitalized dentine in the bottom of a cavity rather than destroy the pulp, but the result was a succession of abscesses and other disagreeable conditions. He said: In these later days I remove everything, regardless of pulp exposure, especially from the bottom of the cavity. If the pulp is that near exposure, I destroy and remove it and fill the pulp-canals, and I am happily free from after troubles.

Dr. E. G. Quattlebaum does not destroy the pulp, if he can avoid it by leaving a small quantity of semi-devitalized dentine which he is careful to thoroughly sterilize, covering it with varnish, followed by cement. He very rarely has dental abscess after this treatment, using pure carbolic acid, 95 percent, for sterilizing.

Dr. Cowardin thought that if the pulp was near exposure it would be injured by the carbolic acid, and that sooner or later Dr. Quattlebaum would have a large percentage of diseased pulps.

Dr. Chas. Sill pursues Dr. Quattlebaum's method, but uses hydrogen peroxide instead of carbolic acid.

Dr. L. G. Noel advocates the removal of all diseased dentine, even at the risk of exposing the pulp. But if there is no peridental inflammation, and the pulp is in a healthy condition, having a little softened dentine, properly sterilized, is preferable to destroying the pulp, where there is no response to tapping the tooth. A valuable filling in these cases is a cement composed of one-third hydronapthol powder to two-thirds cement powder. He has used this in cases of actual exposure without any subsequent trouble. In case of peridental inflammation, seal creosote on cotton or spunk in the cavity for a day or two and wait until the inflammation has subsided. If it does not abate in forty-eight hours, arsenic is the only resource.

Dr. B. Holly Smith would not use carbolic acid so near the pulp, as cicatricial tissue would be unfavorable to the restoration of the pulp to normal condition.

Dr. Frank Holland thinks such an application of carbolic acid would not sterilize the dentine, but it would anesthetize the nerve in the pulp and cause its painless death.

Dr. L. G. Noel commended an old method of pulp capping, given by Dr. J. S. King, of Pittsburg—a paste of pure beechwood creosote and oxide zinc, the cavity being first washed out with water and then saturated with the creosote. He had used that method very successfully for twenty years, though he has lately adopted the hydronaphthol, as recommended by Dr. Stowell.

Dr. Frank Holland said he could kill any nerve with three applications of creosote, which is more escharotic than carbolic acid.

Subject passed.

